# The effect of surgical repair of the chest on postural stability among patients with pectus excavatum

**DOI:** 10.1038/s41598-023-50645-9

**Published:** 2024-01-02

**Authors:** Maria Jarosz, Krystian Pawlak, Wojciech Jarosz, Marzena Wiernicka, Aleksander Barinow-Wojewódzki, Rafał Stemplewski

**Affiliations:** 1 Doctoral School, Poznań University of Physical Education, 27/39 Królowej Jadwigi Street, 61-871 Poznan, Poland; 2 Wielkopolska Center of Pulmonology and Thoracic Surgery, 62 Szamarzewskiego Street, 60-569 Poznan, Poland; 3 Department of Medical Biology, Poznań University of Physical Education, 27/39 Królowej Jadwigi Street, 61-871 Poznan, Poland; 4 Department of Musculoskeletal Rehabilitation, Poznań University of Physical Education, 27/39 Królowej Jadwigi Street, 61-871 Poznan, Poland; 5 Department of Digital Technologies in Physical Activity, Poznań University of Physical Education, 27/39 Królowej Jadwigi Street, 61-871 Poznan, Poland

**Keywords:** Anatomy, Diseases, Medical research

## Abstract

Pectus excavatum is the most encountered of chest wall deformities. It may produce respiratory and cardiovascular symptoms, hence surgical repair of this defect is performed. The procedure involving the insertion of metal bars under the sternum (the Nuss procedure) usually brings significant improvement to patients. However, the effect of the repair on the postural stability of patients has not been studied so far. To investigate the problem of patients' stability in the postoperative period male patients with pectus excavatum (n = 21) and healthy controls (n = 22) were included in the study. Using posturography methods, we showed a negative impact of the pectus excavatum repair surgery on patients' postural stability in the first postoperative phase. The centre of pressure displacement parameters used to measure postural stability were lower after the repair for both, the frontal and sagittal plane as well as for the velocity of displacements in the sagittal plane in the double stance with eyes open. Poorer postural stability was also found in patients with pectus excavatum when compared to healthy controls. Our findings may be useful for functional monitoring in the evaluation and surgical management of pectus excavatum patients and also when designing the rehabilitation of patients undergoing the Nuss procedure.

## Introduction

Chest wall deformities occur in 0.01–0.1% of the population and the most encountered is pectus excavatum (PE)—about 90% of such deformities. The incidence is 1 per 400 live births and 5 to 1 male predominance^1,2^. PE is characterized by depression of the lower part of the sternum and the adjacent costal cartilages altogether with a small change in the structure of collagen. In most patients (60%), the defect appears during puberty, when biological development, including skeletal and muscle growth, accelerates rapidly. Children with PE are often tall, with low BMI and abnormal body posture (scoliosis is diagnosed in about 29% of PE children). PE can also produce respiratory and cardiovascular symptoms including exercise intolerance, fatigue, dyspnea, and chest pain^3–6^. Due to the occurrence of these symptoms, as well as for cosmetic reasons related to self-esteem issues, surgical repair procedures are performed to correct the defect^3,7^. Now the preferred surgical repair is the Nuss procedure. It is a minimally-invasive repair technique of PE (MIRPE), whereby one to three curved metal bars are inserted behind the sternum to correct the shape of the anterior chest wall. The bars are left in situ for three years and then removed^8^. Both stages of the MIRPE are referred to as phases 1 and 2, respectively. In the majority of cases, both pediatric and adult patients experience subjective clinical improvement in exercise tolerance after PE repair^9,10^.

PE is often observed in children with muscular disorders, such as in children with Marfan syndrome, Ehlers-Danlos syndrome, osteogenesis imperfecta, or homocystinuria^3,5^. It is also known that patients with PE who are free of such diseases have greater flexibility of some muscles compared to healthy controls^11^. The muscles of the chest which are affected by PE are mainly respiratory muscles. However, respiratory muscles are also involved in postural control^12,13^. In this context, the question is whether PE affects postural stability.

Postural stability (PS) is described as the ability to maintain the vertical projection of the centre of body mass called also the centre of gravity (COG) inside the base of support^14^. Even in unperturbed conditions COG oscillates and is referred to as postural sway. Such sway always occurs during quiet standing because of our vertical posture which is inherently unstable due to relatively high COG and small base of support^15^. Displacements of COG are transmitted to the support surface as a compound measure of the centre of pressure (COP). In force-plate posturography, trajectories of COP that contain combined information on COG displacements and muscle activity connected to postural reflexes are used to assess postural stability^16,17^.

Postural defects connected with deformation of the body morphology may negatively influence the sensorimotor control of posture and decrease PS. So far, the influence of other than PE posture defects for PS has been studied. Scoliosis has been analyzed in this context. Shreds of evidence for decreased postural stability in scoliotic patients were found—recently reviewed by Dufvenberg et al.^18^. To the authors’ best knowledge this problem has not been analyzed in patients with PE yet. Therefore, the study aimed to determine the effect of MIRPE intervention on the PS of patients. Considering the significant change in the shape of the anterior chest wall due to PE repair, as well as the presence of metal bars in the chest, it was hypothesized that in the short term (phase 1 of MIRPE) the surgical intervention would negatively affect their postural stability. The additional aim was to estimate the difference in postural stability between patients with PE and healthy controls. Taking into account impaired performance of the trunk muscles in PE and reports on the negative impact of other posture defects on postural stability it was hypothesized that postural stability is worse in PE patients than in healthy young men.

## Methods

### Trial design

**Figure 1:**
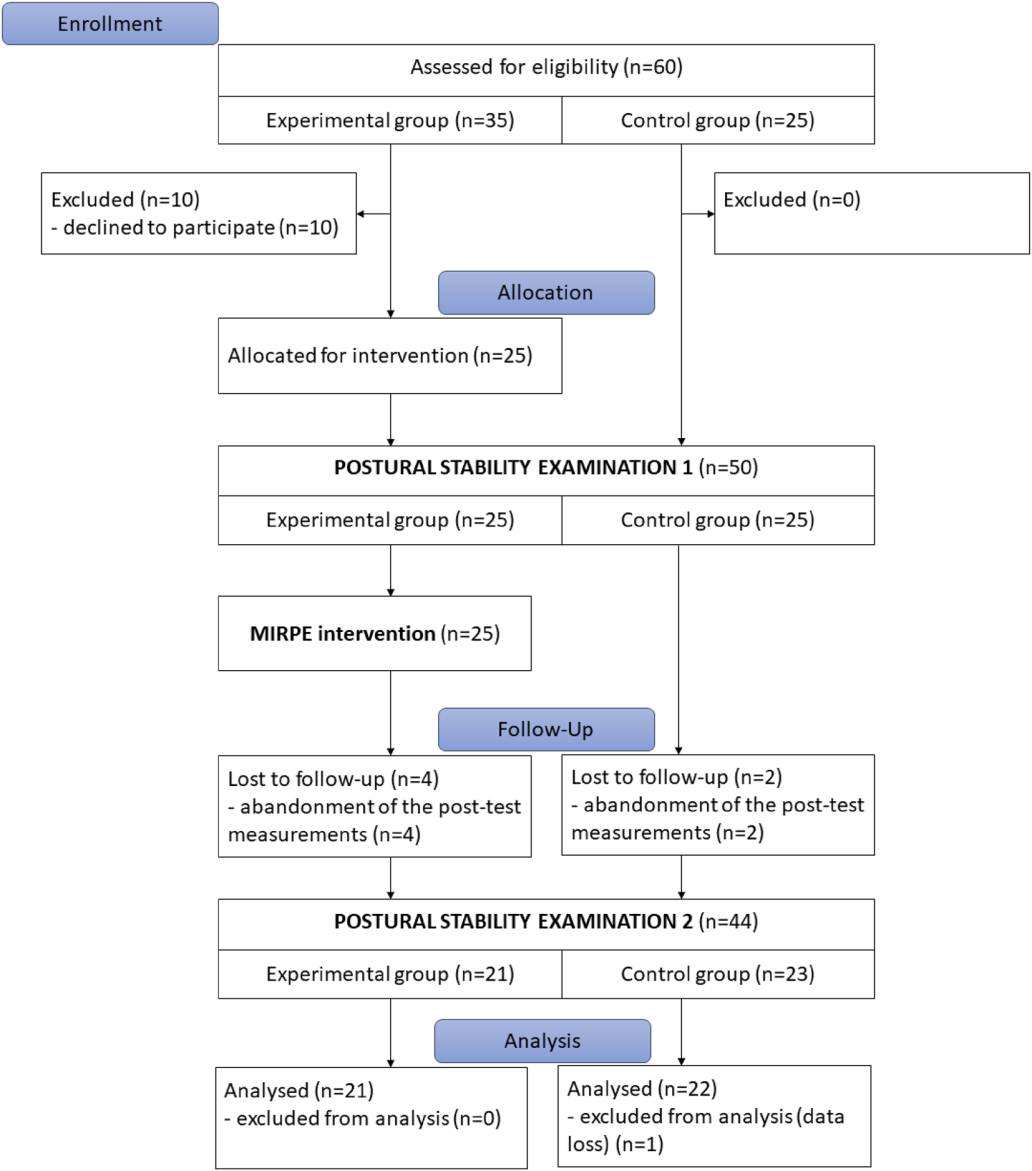
Flowchart of participants and the study design.

The prospective study was designed as a pretest–posttest control trial, as it enables detailed comparisons of within-group time factor (investigating the effect of the intervention) and between groups factor, as well as the cumulative interaction effect of the factors. The clinical examination and surgery repair of selected PE patients—MIRPE were done at Wielkopolska Centre of Pulmonology and Thoracosurgery in Poznań, Poland. Other PS assessments of both, experimental group and control group subjects were done in the Poznań University of Physical Education in Poland. The measurements were carried out twice, before and three months after the MIRPE for the experimental group, and twice for the control group as well (Fig. 1). Before the experiment initial measurements connected to basic and somatic characteristics assessments were recorded. Participants were also familiarized with measurement methods.

### Participants

A total of 35 male patients with PE scheduled for the MIRPE surgery were invited to participate in the study. The inclusion criteria were: a certain age (> 14 years old), no neurological disorders, and consent to the MIRPE surgical procedure. The decisive criterion to exclude a participant from the test group was the co-existence of other defects in the anterior chest wall and the presence of musculoskeletal anomalies (e.g., Marfan syndrome, connective tissue disorders). After excluding patients who refused to participate in the study, 25 participants were included. After MIRPE 4 of 25 patients were lost to follow-up due to the abandonment of post-test measurements. The final 21 patients completed the same measurements 3 months after MIRPE and were included in the experimental group (EG). The recruitment, trials, and measurements of the EG were carried out between 04.2011 and 08.2013.

For comparison (control group—CG) 25 healthy male participants were recruited after fulfilling the same inclusion criteria as for the EG but had neither PE nor another postural defect. Two participants were lost to follow-up due to the abandonment of post-test measurements and one was lost due to the data loss. Finally, 22 healthy participants completed both measurement sessions and were included in the CG.

Subjects were informed in detail about the study procedures. The informed consent was obtained from all subjects (expressed by legal guardians in the case of minors). Participation in the experiment was voluntary. The study was approved by the Bioethics Committee of the Poznań Medical Chamber and was in line with the Helsinki Declaration^19^. The study was retrospectively registered at ClinicalTrials.gov (NCT05844800–06/05/2023).

### Intervention

All PE patients included in the study after preoperative examination underwent the Nuss surgery procedure (MIRPE). The operation was performed under general anesthesia in a hospital setting described in detail previously^20^. Briefly, the surgery involved introducing appropriately bent correctional steel bars (BBH Mikromed, Poland), using video-assisted thoracic surgery through 2 small symmetrical incisions. The implants were introduced retrosternally. To avoid bars displacement, an additional stabilizing bar was introduced, usually at the end of 1 bar, and attached to the adjacent ribs using absorbable sutures^20^.

### Primary outcomes

Postural stability as a dependent variable was examined with the use of the posturography method based on the measurement of COP displacements.

#### Instrumentation and signal processing

The stabilometric platform CQStab2P in a two-plate version(CQ Electronic System, Poland) was used for collecting COP data during trials. The platforms were equipped with strain gauges that facilitated the monitoring of the changes in ground reaction forces. It was connected to a computer equipped with software provided by the manufacturer of the platforms. Based on the data of ground reaction forces the position and displacements of COP were estimated in the software.

The sampling frequency of 200 Hz was used during data acquisition. According to the producer declaration processing accuracy is equal to 0.1% (12-bit processing, effective 10-bit) during the reproduction of statokinesiogram with an accuracy of 1 mm with radius fluctuation of 10 cm^21^.

#### Trials and outcomes

The force platforms were placed on a hard and flat floor surface. Before the start of the testing procedures, the participants rested in a sitting position for 5 min. During the measurement, only the researcher and the participant were present in the room. Participants needed to perform trials in three conditions:

1. Double stance with eyes open (EO);
2. Double stance with eyes closed (EC);
3. One leg standing with eyes open (OLS).

Each trial was run two times—summing 6 trials in each measurement with a 20-s break between the following trials. The order of trials was random to avoid potential learning effects. An average of two repetitions of specific trials was taken as the final result.

The subjects were asked to stand barefoot on the force platforms and stay still with the upper limbs held down the side of the body. For double stance, the feet were placed in a position similar to their natural stance—about 30 degrees to each other^22^. In the case of OLS participants placed the dominant foot in the center of one platform—the same for all participants.

The primary outcomes of the study were:

- Average velocity of COP displacements and its components in anterior–posterior (AP) and mediolateral (ML) directions (Vavg, VavgAP, VavgML, respectively). It’s calculated as a ratio of the total path length covered by COP during the trial, and the time of the trial (mm/s).
- Indicators of the spatial distribution of COP displacements i.e. sway area (SA) and maximal COP displacement in AP and ML directions (MaxAP and MaxML, respectively). The SA indicator is calculated as the size of the area covered by the COP during the trial (mm^2^), while MaxAP and MaxML are calculated as the maximal sway distance (mm) of the COP from the 0.0 point along the Y and X axis in the Cartesian coordinate system, respectively.

Average velocities, maximal displacement, and sway area of COP displacements are commonly used posturographic indicators of postural stability^23^. The average results of the two trials are sufficient to obtain values of the Intraclass Correlation Coefficient above 0.9, at least in the case of the average velocity of COP displacements^24^. As the standard interpretation, it was assumed the increased results of velocity and spatial distribution of COP displacement values as the indicators of decline in PS level^25–28^.

### Age and somatic variables

The study population was characterized by age, body weight and height, and body mass index (BMI)—calculated as body weight/height^2^.

### Statistical analysis

The main calculations related to the assessment of the variability of dependent variables were done based on the ANOVA variance analysis method (test F). Variables were checked for the normality of distribution with the Shapiro–Wilk test. It was found that distribution didn’t differ significantly from the normal one in most of the cases. However, an analysis of variance is quite robust for violation of the normality condition^29^. Taking into account two repeated measurements the condition of sphericity wasn’t relevant.

The analysis was applied taking into account the within-group factor of repeated measurements “time” with two levels (pre and post), and the between-group factor “group” (experimental and control). For interaction effects (“group” × ”time”) the eta-squared effect size was calculated. The effect size indicates the percent of variance explained by particular effects of the dependent variable. To compare the average values of average velocities, area, and maximal COP displacement (both pre-post values within groups, and between groups in pre and post conditions) Bonferroni detailed post-hoc comparisons were used.

Between-group comparisons for age and somatic characteristics in the pretest were done with the use of the t-Student test. The minimum level of statistical significance was defined as p ≤ 0.05. The study was conducted using the Statistica v. 13.0 software program (TIBCO Software Inc., Palo Alto, CA, USA).

## Results

### Results of initial measurements

Basic characteristics for age and somatic parameters are shown in Table 1.

**Table 1:** Average values, standard deviations, minimum, maximum, and 95% confidence intervals for age, and somatic characteristics in experimental and control groups.

	EG (n = 21)			CG (n = 22)			t-Student test (p)
	${\overline{\text{x}}} \pm {\text{SD}}$	Min–max	95% CI	${\overline{\text{x}}} \pm {\text{SD}}$	min–max	95% CI	
Age (years)	16.7 ± 1.45	14.1–19.6	16.0–17.3	17.16 ± 1.38	15.8–21.1	16.5–17.8	1.13 (0.267)
Height (cm)	180.0 ± 7.45	165–200	176.6–183.3	180.1 ± 5.20	170–190	177.8–182.4	0.09 (0.925)
Weight (kg)	61.9 ± 5.57	51.7–70.6	59.3–64.4	79.5 ± 11.34	61.9–101.4	74.5–84.6	6.44 (0.000)
BMI (kg/m^2^)	19.1 ± 1.68	16.0–23.4	18.4–19.9	24.5 ± 2.98	20.1–31.3	23.1–25.8	7.20 (0.000)

*EG* experimental group, *CG* control group, *BMI* body mass index.

Participants from both groups were characterized by similar average body height. On the other hand, higher values of weight and BMI were observed in CG (79.5 ± 11.34 kg and 24.5 ± 2.98 kg/m^2^, respectively) than in EG (61.9 ± 5.57 kg and 19.1 ± 1.68 kg/m^2^, respectively).

### Results of postural stability measurements

**Figure 2:**
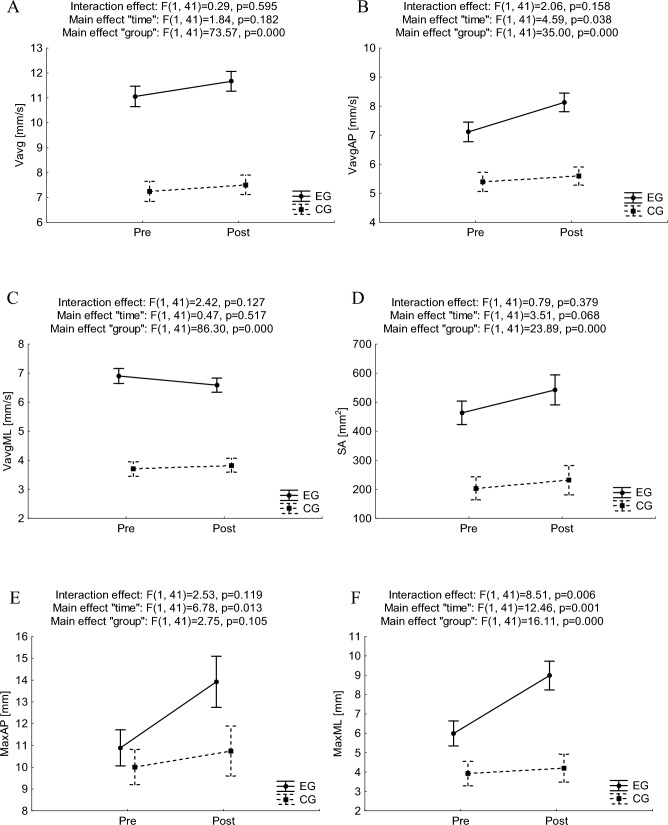
Mean and standard error of measurement values of postural stability indices in double stance with eyes open for pre- and post-trials in experimental and control groups, and results of two-way ANOVA with repeated measures (**A**, **B**, **C**, **D**, **E**, **F** for Vavg, VavgAP, VavgML, SA, MaxAP, MaxML, respectively). *Vavg* average velocity, *SA* sway area, *Max* maximal centre of pressure displacement, *ML* medio-lateral, *AP* anterior–posterior, *EG* experimental group, *CG* control group.

Two-way ANOVA was performed to compare the pretest and posttest values of postural stability indicators of each group (EG and CG), as well as to compare between-group values obtained during trials in three different conditions. Results for ANOVA with repeated measures for PS indicators in trials of EO are presented in Fig. 2.

There was no statistically significant interaction effects “time” × ”group” observed for average velocities. On the other hand, in the case of Vavg, VavgAP, and VavgML there were found significant main effects of “group” (F_(1,41)_ = 73.57, p = 0.000, ƞ^2^ = 0.64; F_(1,41)_ = 35.00, p = 0.000, ƞ^2^ = 0.46 and F_(1,41)_ = 86.30, p = 0.000, ƞ^2^ = 0.68, respectively) with higher average values in EG (which might be interpreted as worse PS) than in CG, both for pretest and posttest (Bonferroni post-hoc analysis—at least p < 0.01). It was also observed the main effect of “time” for VavgAP (F_(1,41)_ = 4.59, p = 0.038, ƞ^2^ = 0.10) but with no clear simple effect of “time” in both EG (p = 0.099) and CG (p = 1.000).

In the case of SA no statistically significant interaction effects “time” × ”group” was observed, yet a clear main effect of “group” was noticed (F_(1,41)_ = 23.89, p = 0.000, ƞ^2^ = 0.37) with higher values in EG than in CG, both in pretest (p < 0.01) and posttest (p < 0.001). There was also a statistically significant “time” effect for MaxAP (F_(1,41)_ = 6.78, p = 0.013, ƞ^2^ = 0.14) as a consequence of higher values in the posttest than in the pretest in EG (p = 0.033), which might be interpreted as worse PS after the intervention. Statistically significant interaction effect “time” × ”group” (F_(1,41)_ = 8.51, p = 0.006, ƞ^2^ = 0.17) was observed for MaxML. The posttest value in EG was higher in comparison to the pretest value in EG (p < 0.001) as well as in comparison to the posttest value in CG (p < 0.001), which might be interpreted as worse PS in EG. Also for MaxML the main effect of “time” (F_(1,41)_ = 12.46, p = 0.001, ƞ^2^ = 0.23), and the main effect of “group”(F_(1,41)_ = 16.11, p = 0.000, ƞ^2^ = 0.28) for MaxML were noticed.

**Figure 3:**
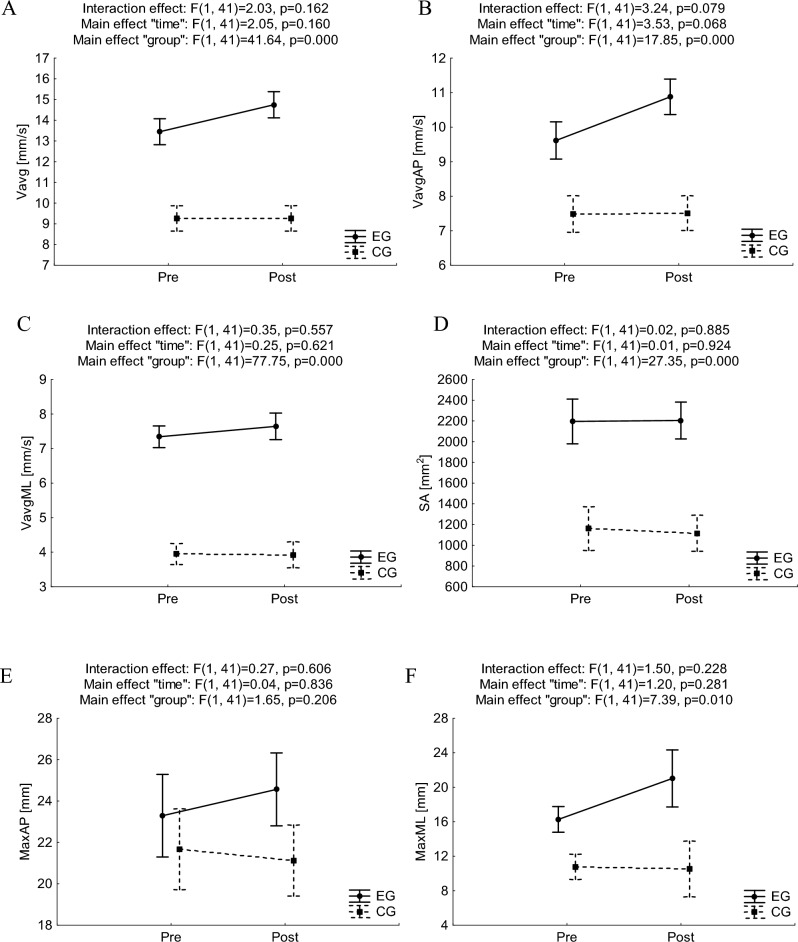
Mean and standard error of measurement values of postural stability indices in double stance with eyes closed for pre- and post-trials in experimental and control groups, and results of two-way ANOVA with repeated measures (**A**, **B**, **C**, **D**, **E**, **F** for Vavg, VavgAP, VavgML, SA, MaxAP, MaxML, respectively). *Vavg* average velocity, *SA* sway area, *Max* maximal centre of pressure displacement, *ML* medio-lateral, *AP* anterior–posterior; *EG* experimental group, *CG* control group.

Results for ANOVA with repeated measures for postural stability indicators in trials of EC are presented in Fig. 3.

In the case of Vavg, VavgAP, and VavgML no statistically significant interaction effects “time” × ”group” was observed, however significant main effects of “group” were noticed (F_(1,41)_ = 41.64, p = 0.000, ƞ^2^ = 0.50; F_(1,41)_ = 17.85, p = 0.000, ƞ^2^ = 0.30 and F_(1,41)_ = 77.75, p = 0.000, ƞ^2^ = 0.65, respectively). Bonferroni's detailed post-hoc analysis revealed higher values in EG than in CG, both in the pretest (at least p < 0.05) and the posttest (p < 0.001), which might be interpreted as worse PS in EG.

A statistically significant main effect of “group” was also observed for SA (F_(1,41)_ = 27.35, p = 0.000, ƞ^2^ = 0.40) with higher values in EG than in CG in the pretest and posttest (p < 0.001), and for MaxML (F_(1,41)_ = 7.39, p = 0.010, ƞ^2^ = 0.15) with higher values in EG than in CG in posttest (p < 0.05), which might be interpreted as worse PS in EG. No statistically significant interaction effects “time” × ”group” were observed for the spatial distribution of COP displacement indicators.

**Figure 4:**
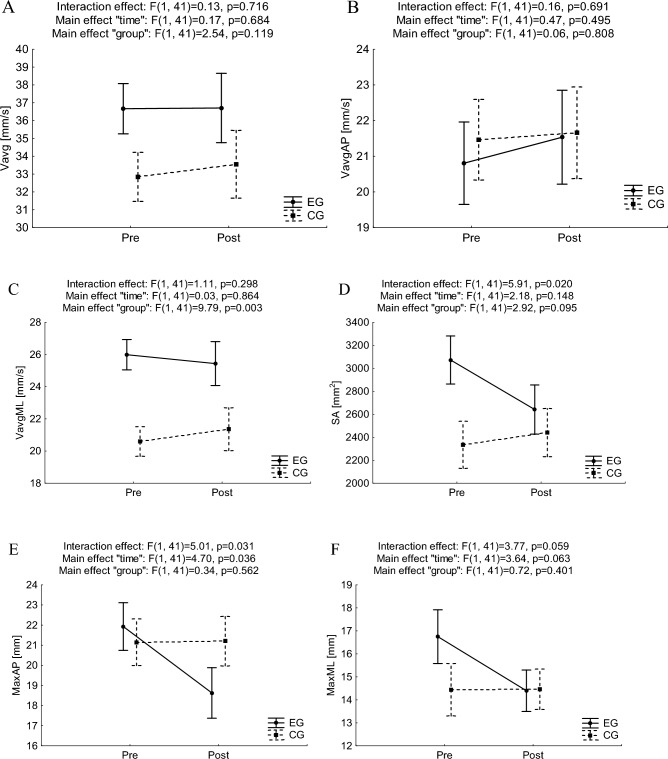
Mean and standard error of measurement values of postural stability indices in one-leg stance with eyes open for pre- and post-trials in experimental and control groups, and results of two-way ANOVA with repeated measures (**A**, **B**, **C**, **D**, **E**, **F** for Vavg, VavgAP, VavgML, SA, MaxAP, MaxML, respectively). *Vavg* average velocity, *SA* sway area, *Max* maximal centre of pressure displacement, *ML* medio-lateral, *AP* anterior–posterior, *EG* experimental group, *CG* control group.

Results for ANOVA with repeated measures for postural stability indicators in trials of OLS are presented in Fig. 4.

No statistically significant interaction effects “time” × ”group” were observed for both, velocities and spatial distribution of COP displacement indicators in one leg standing with eyes open trials.

In the case of velocities of COP displacements, there was observed a statistically significant main effect of “group” only for VavgML (F_(1,41)_ = 9.79, p = 0.003, ƞ^2^ = 0.19) with higher values in EG than in CG in the pretest (p < 0.05).

In the case of PS indicators connected to the spatial distribution of COP displacements, there were found statistically significant interaction effects “time” × “group” for SA and MaxAP (F_(1,41)_ = 5.91, p = 0.020, ƞ^2^ = 0.13 and F_(1,41)_ = 5.01, p = 0.031, ƞ^2^ = 0.11, respectively). Bonferroni's detailed post-hoc analysis revealed slightly lower values in the posttest than in the pretest for SA (difference didn’t reach statistical significance (p = 0.055), and MaxAP (p = 0.022). Also, a main effect of “time” for MaxAP was noticed (F_(1,41)_ = 4.70, p = 0.036, ƞ^2^ = 0.10).

The average values and baseline statistics of all measured indicators of postural stability for both, the experimental and control group, as well as differences between pretest and posttest values, for all three trial conditions are shown in Table 2 (supplementary material).

## Discussion

The MIRPE procedure brings patients improvement, especially in pulmonary function, cardiac function, exercise tolerance as well as in self-esteem. However, many authors point to ambiguous data on the effects of the surgery^30–32^. The main reason for the conflicting observations is the moment of examining the patients, i.e. the different time that has elapsed since the metal bars were inserted (or removed) in the chest. It has been observed that the early postoperative phase may be associated with a decrease in respiratory parameters, which improve over time, especially after removing the bars^30^. The first three months after surgery (bars insertion—phase 1) is a period of recovery, increased pain (especially the first weeks), and, consequently, a reduction of physical activity. This may be crucial for the decrease in many physiological parameters, not only cardiorespiratory but also the performance of muscles, which may also affect PS. After 3–5 months from the surgery, a gradual return to full physical activity is possible^33,34^. So, phase 1 and phase 2 outcomes cannot be considered together.

In this study, patients were examined before and three months after the MIRPE intervention (phase 1). In the first such study, it was found that the PS of PE patients deteriorates after surgical repair as indicated by higher averages of parameters measuring the velocity of COP displacements as well as indicators of the spatial distribution of COP displacements. A statistically significant effect of the time was observed in the case of maximal COP displacement in the frontal plane (MaxML) and sagittal plane (MaxAP) as well as the velocity of COP displacements in the sagittal plane (VavgAP) in the double stance with eyes open. With eyes closed, the results were similar, although neither equally strong effects, nor significant effects were noted.

Different results were obtained for the one-leg stance with eyes open test. On the one hand, a significant decrease in PS was not confirmed when comparing the results before and after the MIRPE intervention. On the other hand, in the case of the parameters of the spatial distribution of COP displacement (SA, MaxAP, and MaxML), lower average values were observed after the MIRPE, and although the differences were not statistically significant, the opposite trend was observed than in the case of the double stance trials. Perhaps there was an effect of muscle stiffness, which increases as the risk of falling increases. It is known that stiffness, including the area of the trunk, increases the likelihood of a loss of balance^35–37^. It can be presumed that after surgery patients are accompanied by a greater fear of falling, which is related to greater pain and uncertainty of feeling their own body at that time. This may result in greater mobilization during a difficult attempt at one-leg standing. However, this requires further research, extended by other measurements, such as electromyography.

The effect of MIRPE on PS should be considered during the recovery phase. Its better course is ensured by properly selected physiotherapy. Especially in the first phase after the surgery, it is recommended to include exercises and physiotherapeutic care. Strengthening the trunk muscles improves body posture and the general condition of the body, especially in the area of the correction made^38^. Goretsky et al.^39^ argue that exercise programs are as important as the surgery itself in leading to full chest function and associated full fitness, mainly aerobic capacity. Decreased PS in the first phase after surgery should be taken into account when designing a rehabilitation program, as the physiological impact of intense physical activity is also responsible for a range of acute fatigue effects on the neuromotor system that can adversely impact postural control^40^. While in healthy people with proper balance this is not a problem, in the case of patients with poorer stability, as of patients in the first phase after the MIRPE procedure, it is necessary to introduce such exercises and therapy that will counteract the negative effects of a decreased PS.

When comparing the PS of PE patients with healthy controls, higher values of PS indicators were generally observed in PE subjects. Both, parameters measuring the velocity of COP displacement (Vavg, VavgAP, and VavgML) and spatial distribution of COP displacement (SA) reached significantly higher mean values in EG than in CG (pretest). This applies to both the double stance with eyes open and with eyes closed. In the case of the one-legged position, all analyzed PS indicators, apart from VavgAP, were on average higher in the experimental group, but a significant difference between EG and CG (pretest) was observed only for the average values of the VavgML parameter. The results of the PS measurements comparison mean that patients with PE are characterized by greater COP displacement or postural sway in the quiet stance, and thus worse PS than healthy controls. Differences between PE and healthy individuals may be due to several issues. One of them is body morphology. The difference between the PE patients and the control group in terms of basic somatic characteristics was significantly lower body weight with similar body height. This translates into a significantly lower BMI in PE. This is not surprising as PE is often associated with asthenic physique^41^. Previous reports on the relationship between BMI and PS do not show unequivocal results. On the one hand, postural control is found to be negatively correlated with increased adiposity, as the obese BMI group performance is significantly poorer than the underweight, normal weight, and overweight groups during double and single-leg stance^42^—similar results were also obtained by other authors^43–46^. On the other hand, in the study by Błaszczyk et al.^47^ heavier patients presented a significant reduction in postural sway. The main parameters of postural sway, i.e. the total path length and its directional components, were negatively correlated with body weight and BMI^47^. It can be presumed that not only BMI, but many anthropometric factors are important for PS. Their interaction, which will produce different results in the case of very thin and obese, short and tall individuals, also translates into differences in PS between them. In conditions accompanied by weight change, musculoskeletal factors seem to play a major role in reduced PS, which appears to be related to weight fluctuations, rather than absolute BMI values as inferred from studies of patients with bulimia. In the case of anorexic patients, on the other hand, there was no clear postural instability compared to healthy patients^48^. Alonso et al.^49^ found that postural control depends on body composition as well as on body dimension and this relation is mediated by the sensory information. They showed that height was the anthropometric variable that most influenced the postural sway. However, in this study subjects from EG and CG had similar heights, so other factors were crucial for the difference between groups.

Impairment of muscle function related to the abnormal structure of the anterior chest wall in patients with PE translates not only into the deterioration of the respiratory mechanism but also may affect postural stability. It is known that all respiratory muscles are simultaneously involved in postural control^12,13^. This applies to basically all the muscles that originate or insert onto the trunk. It means that external and internal forces that affect the function of the respiratory muscles will also affect postural responses^13^. It has been previously shown that the flexibility of muscles such as the pectoralis major and the internal rotators of humeral joints is greater in PE patients^11^. So it may be an important factor leading to a decrease in PS among PE patients when compared to healthy controls^41,50^.

## Conclusions

Studies on the impact of posture defects on PS are not excessively numerous, especially if they concern changes caused by a surgical procedure performed to correct the defect. Furthermore, they are carried out according to different protocols and on different posturographic platforms, which means that comparing the results of these tests is not always fully possible. Based on the results of this study, the hypothesis about the negative impact of PE repair surgery on patients' PS in the first postoperative phase might be confirmed. However, it should be noted that the study group was relatively small and although more conservative post-hoc statistical analyses were used, our interpretations and conclusions remain temperate. This issue requires further in-depth research on larger cohorts as well as over a longer time frame. An extended examination period would allow researchers to determine the dynamics of PS changes after the end of the entire PE repair procedure, related to full convalescence, fixation of the desired changes in the structure of the anterior chest wall, and removal of metal bars. In addition, a deeper understanding of PS could provide a supplementary diagnostic tool for functional monitoring in the evaluation and surgical management of PE patients. Obtained results indicating a possible decrease in PS in the first phase after surgical intervention should be taken into account when designing the rehabilitation of patients undergoing the MIRPE procedure.

The second objective of the study was to compare PE patients with healthy controls for PS. The obtained results indicate a poorer PS in the double stance position of patients with this postural defect. Continuation of posturographic research will contribute to a better understanding of the complex and complicated mechanism of functioning of the chest in people with PE, which includes not only postural control but also the issues of proper interaction of muscles and other elements of the trunk in the precise regulation of the breathing mechanism.

## Limitations

Our study has some limitations worth noting. We had a relatively small sample size. The experimental group consisted of 21 PE patients who completed the trials before and after MIRPE. The other limitation was the length of follow-up. The effect of MIRPE on PS was tested only for the 1 phase of the PE repair surgery. In subsequent studies, it is worth considering the inclusion of such issues as the assessment of physical activity level, as it is known that physical activity can modulate the posture control of people of all ages, and low level of physical activity can be responsible for insufficient posture control^50^, as well as assessment of muscle function (e.g. EMG) and postural alterations typical in PE patients.

### Supplementary Information

Supplementary Information.

Supplementary Table 2.

Supplementary Legends.

## Data Availability

All data generated or analyzed during this study are included in this article (and its Supplementary Information files).
