# Supplementary material for: The effect of surgical repair of the chest on postural stability among patients with pectus excavatum
Source: Sci Rep. 2024 Jan 2;14:45. doi: 10.1038/s41598-023-50645-9 (PMC10762140; doi:10.1038/s41598-023-50645-9)
Supplement: Supplementary file 3 — Supplementary Legends. [file 41598_2023_50645_MOESM3_ESM.docx]

**Table 2.** (supplementary material) The average values and baseline statistics of all measured indicators of postural stability for both, experimental and control groups, as well as differences between pretest and posttest values, for all three trial conditions
